# Supplementary material for: Clinical, Serologic, and Histologic Characteristics in Screen-Detected and Clinically Diagnosed Celiac Disease Patients
Source: Gastro Hep Adv. 2026 Apr 17;5(7):100973. doi: 10.1016/j.gastha.2026.100973 (PMC13200102; doi:10.1016/j.gastha.2026.100973)
Supplement: Supplementary Tables 1 and 2 [file mmc1.pdf]

Supplementary Table 1. Presence of co-morbidities in 214 celiac disease patients identified by screening or in clinical practice.

	Screen-detected n=102	Symptom-detected n=112	*P* value
	%	%	
Asthma	6	6	1.000
Coronary artery disease	5	5	1.000
Hypertension	16	10	.303
Lactose intolerance	11	11	1.000
Malignancy[1]	6	4	.529
Neurological disorder[2]	4	1	.202
Other autoimmune disease[3]	8	4	.242
Psoriasis	5	1	.111
Psychiatric disorder[4]	5	6	1.000
Thyroidal disease	15	18	.581
Type 1 diabetes	3	2	.676

[1]Breast cancer, lymphoma, adenocarcinoma, prostate carcinoma, ovarian carcinoma, ventricular carcinoma, basal cell carcinoma, melanoma; [2]Hemiplegia, polyneuropathy, benign paroxysmal positional vertigo, cerebral infarction; [3]Sjögren's syndrome, IgA nephropathy, IgA deficiency, systemic lupus erythematosus, alopecia, rheumatoid arthritis; [4]Depression, panic disorder, obsessive-compulsive disorder, vital exhaustion.

Supplementary Table 2. Clinical characteristics at diagnosis, and symptoms and health-related quality of life at diagnosis and after one year of treatment in 102 screen-detected celiac disease patients with or without self-reported symptoms at diagnosis.

	Asymptomatic n=57	Symptomatic n=45	*P* value[1]
	%	%	
Females	47	53	.690
Family history of celiac disease	67	74	.511
Strict GFD adherence	91	98	.225
	Median (Q1, Q3)	Median (Q1, Q3)	
Age, years	53 (37, 62)	54 (43, 59)	.701
TGA, U/mL	18.3 (7.3, 47.7)	28.6 (11.3, 76.0)	.263
EmA, titer	1:200 (1:50, 1:500)	1:200 (1:50, 1:500)	.681
VH/CrD	0.3 (0.1, 0.9)	0.2 (0.1, 0.8)	.524
IELs, /100 epithelial cells	52.1 (37.8, 64.5)	49.8 (35.4, 65.2)	.949
Hemoglobin, g/dL	140 (132, 148)	140 (131, 153)	.363
Lumbar T-score	-1.0 (-2.0, 0.0)	-0.4 (-1.4, 1.2)	**.018**
Femoral T-score	-0.9 (-1.9, -0.1)	-0.6 (-1.5, 0.0)	.287
BMI, kg/m^2	24.6 (22.4, 28.0)	24.3 (22.7, 28.5)	.066
GSRS, total score			
At diagnosis	1.6 (1.3, 2.4)	2.2 (1.6, 2.6)	**.011**
On GFD	1.4 (1.1, 1.7)[1]	1.6 (1.2, 1.8)[1]	.080
PGWB, total score[2]			
At diagnosis	108 (101, 117)	107 (89, 113)	.305
On GFD	113 (105, 119)[3]	110 (100, 119)	.312

[1]Significant improvement on GFD, *P*<.001. [2]No significant differences in sub-scores between the groups at diagnosis or on GFD. [3]Significant improvement on a GFD, *P*=.015. BMI, body mass index; EmA, endomysial antibodies; GSRS, Gastrointestinal Symptom Rating Scale, higher scores denote more symptoms; IELs, intraepithelial lymphocytes; PGWB, Psychological General Well-Being questionnaire, higher scores denote better quality of life; TGA, transglutaminase 2 antibodies; VH/CrD, villous height-crypt depth ratio.
