# Supplementary material for: Clinical, Serologic, and Histologic Characteristics in Screen-Detected and Clinically Diagnosed Celiac Disease Patients
Source: Gastro Hep Adv. 2026 Apr 17;5(7):100973. doi: 10.1016/j.gastha.2026.100973 (PMC13200102; doi:10.1016/j.gastha.2026.100973)
Supplement: Extended PDF [file mmc2.pdf]

Gastro Hep Advances 2026;5:100973

ORIGINAL RESEARCH—CLINICAL

Clinical, Serologic, and Histologic Characteristics in Screen-Detected and Clinically Diagnosed Celiac Disease Patients

Iida Ahonen,[1] Laura Kivelä,[1,2,3] Aku Paavola,[1] Pilvi Laurikka,[1,4] Heini Huhtala,[5] Harri Sievänen,[6] Katri Kaukinen,[1,7] and Kalle Kurppa[1,3,8,9]

[1]Tampere Celiac Disease Research Center, Faculty of Medicine and Health Technology, Tampere University, Tampere, Finland; [2]Children's Hospital and Pediatric Research Center, University of Helsinki and Helsinki University Hospital, Helsinki, Finland; [3]Department of Pediatrics, Tampere University Hospital, Wellbeing Services County of Pirkanmaa, Tampere, Finland; [4]Department of Internal Medicine, Kanta-Häme Central Hospital, Hä;meenlinna, Finland; [5]Faculty of Social Sciences, University of Tampere, Tampere, Finland; [6]The UKK Institute for Health Promotion Research, Tampere, Finland; [7]Department of Internal Medicine, Tampere University Hospital, Wellbeing Servicers County of Pirkanmaa, Tampere, Finland; [8]The University Consortium of Seinäjoki, Seinäjoki, Finland; and [9]Department of Pediatrics, Seinäjoki Central Hospital, Seinäjoki, Finland

BACKGROUND AND AIMS: The benefits of celiac disease screening remain debatable. We compared baseline characteristics and treatment outcomes between screen-detected and clinically diagnosed celiac disease adults in a prospective study. **METHODS:** Clinical, serologic, and histologic data and bone mineral density (BMD) were assessed in 214 patients at diagnosis and after 1 year on gluten-free diet (GFD). Validated questionnaires were used to elicit gastrointestinal symptoms (Gastrointestinal Symptoms Rating Scale) and quality of life (Psychological General Well-Being questionnaire). **RESULTS:** Screen-detected patients (n = 102) were older (median, 54 vs 43 years; $P = .005$), more often men (50% vs 16%; $P < .001$), and had higher mean lumbar T-score (−0.8 vs −1.2 standard deviation; $P = .011$) and hemoglobin levels (140 vs 128 g/dL; $P < .001$) at diagnosis than clinically diagnosed patients (n = 112). The groups were comparable in transglutaminase 2 (24.5 vs 47.7 U/mL; $P = .051$) and endomysial (1:200 vs 1:200; $P = .385$) autoantibody levels and histology assessed by villous height/crypt depth ratio (0.3 vs 0.3; $P = .964$). Screen-detected patients had fewer gastrointestinal symptoms (Gastrointestinal Symptoms Rating Scale total score 1.9 vs 2.6; $P < .001$) and better quality of life (Psychological General Well-Being questionnaire total score 107 vs 98; $P = .001$). After 1 year, both groups showed similar GFD adherence (94% vs 95%; $P = .000$) and improvements in histology, BMD, and quality of life. Asymptomatic and symptomatic screen-detected patients were comparable in histologic findings at diagnosis, adherence to GFD, and improvements in symptoms and quality of life. **CONCLUSION:** Screen-detected celiac disease patients demonstrated less severe symptoms and BMD impairment at diagnosis but advanced histologic and serologic disease similar to those identified on clinical grounds. Adherence to GFD and treatment response were comparable between the groups.

Keywords: Celiac Disease; Serology; Screening; Quality of Life; Asymptomatic

Introduction

Celiac disease is a chronic immune-mediated disorder, estimated to affect 1% of the population globally, with prevalence reaching up to 2.4% in Finland.[1,2] However, the majority of patients experience long diagnostic delays or remain unidentified despite increased awareness of the condition and the availability of noninvasive serologic testing.[3,4] This can be explained at least in part by the exceptionally heterogenous clinical presentation, which ranges from asymptomatic to severe gastrointestinal and extra-intestinal manifestations. Additionally, even the gastrointestinal complaints, often considered typical of celiac disease, have poor predictive value for the subsequent diagnosis, further complicating clinical case finding.[1,5]

The low diagnostic efficiency could be enhanced through testing of known at-risk groups, such as family members of patients and individuals with other autoimmune diseases—an approach already recommended by several guidelines.[6–9] In fact, even population-based approaches to screening are emerging.[10] However, the existing evidence regarding the overall benefits of this strategy, particularly in apparently asymptomatic individuals, remains limited.[11,12] While symptomatic patients have an overrepresentation of various complications, such as

Abbreviations used in this paper: **BMD, bone mineral density; BMI, body mass index; EmA, endomysial antibodies; GFD, gluten-free diet; GSRS, Gastrointestinal Symptoms Rating Scale; Hb, blood hemoglobin; IEL(s), intraepithelial lymphocyte(s); PGWB, Psychological General Well-Being questionnaire; TGA, tissue transglutaminase 2 antibodies.**

Most current article

© 2026 The Author(s). Published by Elsevier Inc. on behalf of American Gastroenterological Association Institute. This is an open access article under the CC BY license (http://creativecommons.org/licenses/by/4.0/).
2772-5723
https://doi.org/10.1016/j.gastha.2026.100973

osteoporosis and malignancy,[13] it is less clear whether this also applies to individuals detected through screening, who may have less advanced duodenal damage.[14] Furthermore, there is concern that the treatment—a lifelong gluten-free diet (GFD)—could cause burden, have a negative effect on quality of life, and be potentially poorly accepted, particularly among asymptomatic patients.[15,16]

To further explore these questions, we compared a wide range of clinical and histologic variables, laboratory parameters, bone mineral density (BMD), and quality of life both at diagnosis and after 1 year on GFD between adult patients identified through clinical suspicion and screening. Particular attention was paid to individuals with celiac disease who appeared asymptomatic.

Material and Methods

Patients and Study Design

The study was conducted at Tampere University and Tampere University Hospital. It comprised ≥15-year-old patients who were diagnosed with a biopsy-proven celiac disease between 1998 and 2014 and participated in prospective follow-up. Altogether, 214 patients with newly diagnosed celiac disease met the inclusion criteria. They underwent investigations according to the predefined study design of each series, initiated GFD under the guidance of a dietitian, and had a follow-up visit scheduled after 1 year on the diet. All variables were compared between patients diagnosed through intentional at-risk group or population screening ("screen-detected patients") and those diagnosed by because of clinical suspicion ("clinically detected patients"). Screen-detected patients were tested either due to belonging to a risk group, such as having a family history of celiac disease, having another autoimmune condition, or through participation in a population-based screening study. Clinically detected patients were identified due to symptoms or manifestations related to the disease, including gastrointestinal and extraintestinal complaints, and conditions such as infertility, anemia, or osteoporosis. Also, part of the screen-detected patients reported celiac disease–related symptoms at the time of diagnosis and were therefore further categorized into asymptomatic and symptomatic subgroups.

Ethical Aspects

The Regional Ethics Committees of Tampere University Hospital and the Pirkanmaa Hospital District approved the study protocol and patient recruitment (ethical committee codes E98012, R03041, and R07122). All participants were informed about the study and provided written informed consent. They were aware of their option to withdraw their consent to participate at any time without this affecting their treatment. The ethical guidelines of the Declaration of Helsinki were strictly followed.

Study Variables

Participants underwent a thorough clinical examination and data collection of demographic factors, clinical presentation, family history of celiac disease, and presence of celiac disease–associated and other chronic comorbidities. Self-perceived gastrointestinal symptoms and health-related quality of life were further elicited using specific questionnaires (see below). Additionally, blood was drawn for celiac disease serology and other laboratory measurements, and esophagogastroduodenoscopy with systematic duodenal biopsies, along with BMD measurement, were conducted in accordance with the study design. The baseline evaluations were repeated after 1 year on GFD.

Serum endomysial antibodies (EmA) were determined by indirect immunofluorescence.[17] A dilution of 1: ≥5 for EmA was considered positive and further diluted up to 1:4000 or until negative. Serum transglutaminase 2 antibodies (TGA) were measured using a commercial assay (Celikey; Phadia, Freiburg, Germany). The cutoff for TGA positivity was set at ≥3.0 U.[18] Additionally, depending on the design of each study, the following celiac disease–related laboratory values were obtained using routine methods: blood hemoglobin (Hb; reference range: men, 13.4–16.7 g/dL; women, 11.7–15.5 g/dL), erythrocyte folic acid (200–700 nmol/L), and serum vitamin B12 (150–740 pmol/L).

A minimum of 6 representative mucosal samples was routinely taken from the duodenum using biopsy forceps during esophagogastroduodenoscopy. The degree of mucosal inflammation, defined as microscopically counted number of intraepithelial lymphocytes (IELs)/100 epithelial cells, and the quantitative villous height/crypt depth ratio were assessed from well-oriented biopsy specimens. Celiac disease diagnosis was set by the hospital pathologist based on the presence of mucosal inflammation and morphological damage (ie, ≥Marsh-Oberhuber 3a).[19] A portion of the biopsies was snap-frozen in liquid nitrogen for immunohistochemical analyses of mucosal CD3+ and $\gamma\delta$+ IEL densities, following standard operating procedures validated at our study center.[20]

BMD was measured in consenting participants from the lumbar spine (L2-L4) and femoral neck by dual-energy X-ray absorptiometry (Norland XR-26, Norland Corp, WI). The values were expressed as T-scores, which compare individual BMD values to those of young adults of the same sex. T-scores >-1.0 were considered normal, scores between -1.0 and -2.5 osteopenic, and scores <-2.5 indicative of osteoporosis.[21] Body mass index (BMI) was determined as kg/m^2, with values <18.5 defined as underweight, 18.5–24.9 normal, 25.0–29.9 overweigh, and ≥30.0 obese. Adherence to GFD was evaluated through a systematic interview, with the diet considered strict if inadvertent gluten intake occurred only sporadically, that is, a few times a year.

The validated Gastrointestinal Symptoms Rating Scale (GSRS) questionnaire was used to measure the self-estimated severity of gastrointestinal symptoms.[22] This is a 15-item questionnaire that uses a 7-point Likert-scale and provides a numerical score for the severity of 5 different symptom categories, including diarrhea, indigestion, constipation, and abdominal pain and reflux, as well as a total score. Subdimension scores are calculated as means of each relevant item and total score as a mean of all items. A higher score indicates more severe gastrointestinal symptoms.

Health-related quality of life was assessed using the Psychological General Well-Being questionnaire (PGWB), which is a 22-item questionnaire using a 6-point Likert scale.[23] The survey provides a numerical score for quality of life on 6 subdimensions (anxiety, depression, well-being, self-control,

Table 1. Serologic and Histologic Parameters at Celiac Disease Diagnosis and After 1 Year on Treatment in 214 Patients Identified by Screening or in Clinical Practice

Parameters	Screen-detected (n = 102) Median (Q1, Q3)	Clinically detected (n = 112) Median (Q1, Q3)	*P* value[1]	*P* value[2]
TGA, U/mL				
At diagnosis	24.5 (8.0, 64.4)	47.7 (9.9, 99.6)	.051	.145
On GFD	0.2 (0.0, 1.6)[3,5]	1.6 (0.8, 3.3)[3]	**<.001**	.243
EmA, titer				
At diagnosis	1:200 (1:50, 1:500)	1:200 (1:50, 1:500)	.385	.213
On GFD	Negative[3]	Negative[3]	.190	.141
VH/CrD				
At diagnosis	0.3 (0.1, 0.8)	0.3 (0.1, 0.6)	.964	.387
On GFD	2.3 (1.9, 2.9)[3]	2.4 (1.7, 2.7)[3]	.459	.073
IELs/100 epithelial cells				
At diagnosis	51.0 (37.7, 65.1)	43.8 (38.0, 50.5)	**.008**	**.002**
On GFD	29.5 (21.6, 37.8)[3]	25.3 (20.0, 32.9)[3]	**.019**	.083
$\gamma\delta+$ IELs/mm				
At diagnosis	21.0 (13.3, 31.7)	15.7 (11.2, 25.1)[6]	**.010**	**.015**
On GFD	16.7 (10.1, 24.9)[3,7]	10.5 (5.8, 17.8)[4]	**<.001**	**<.001**
CD3+ IELs/mm				
At diagnosis	75.5 (51.3, 95.0)	66.5 (52.8, 82.3)[8]	.069	.056
On GFD	42.3 (32.0, 55.8)[3,9]	35.0 (21.0, 52.8)[3]	**.010**	**.008**

Screen-detected vs clinically detected patients: [1]crude analysis and [2]adjusted for age and sex. At diagnosis vs on GFD, [3]*P* < .001 and [4]*P* = .001. Data were available for >80% of patients, except in [5]78%, [6]64%, [7]60%, [8]59%, and [9]63%. Bolded *P* value denotes statistical significance.
EmA, endomysial antibodies; GFD, gluten-free diet; IELs, intraepithelial lymphocytes; TGA, transglutaminase 2 antibodies; Vh/CrD, villous height/crypt depth ratio.

general health, and vitality) and a total score. Each subscore is calculated as sums of the items on each subdimension. The total score is the sum of all items and ranges from 22 to 132. A higher score indicates better quality of life.

Statistics

Quantitative data were expressed as means and standard deviations for normally distributed parametric data and as medians and interquartile ranges for nonparametric or skewed data. Normal distribution was evaluated visually by histograms and, when needed, with Kolmogorov-Smirnov and Shapiro-Wilk tests. Differences between the study groups were assessed with Student *t*-test, Mann-Whitney *U* test, or Chi-square test as appropriate, and changes within the groups over time with Wilcoxon test. Logistic regression analysis was used to adjust for age and sex difference between screen-detected and clinically detected patients. *P* values $\leq$.05 were considered significant. All analyses were performed with Statistical Package for the Social Sciences statistical software (version 28.0; IBM, Armonk, NY).

Results

Screen-detected patients (n = 102) were significantly older (median 54 vs 43 years; *P* = .005), more often men (50% vs 16%; *P* < .001), and more frequently had 1 or more family member with celiac disease (70% vs 32%; *P* < .001) compared to those identified on clinical grounds (n = 112). The groups did not differ in the presence of comorbidities (Supplementary Table 1). Screening was performed through risk-group screening in 64 (63%) and through population screening in 38 (37%) individuals. Overall, 94% of screen-detected and 95% of clinically detected patients reported adhering to a strict GFD 1 year after diagnosis (*P* = 1.000).

At diagnosis, screen-detected patients had higher densities of routinely counted IELs and $\gamma\delta+$ IELs than did clinically detected patients, whereas immunohistochemically determined CD3+ IEL densities and levels of TGA, EmA and villous height/crypt depth ratio were comparable (Table 1). All serologic and histologic parameters improved significantly in both groups on GFD, although treated screen-detected patients still exhibited higher densities of $\gamma\delta+$ and CD3+ IELs. Additionally, screened patients had lower TGA levels on GFD in crude analysis but not after adjusting for age and sex, compared to symptom-detected patients. Median TGA levels were low in both groups on GFD (Table 1).

Screen-detected patients had higher Hb levels at diagnosis both in crude analysis and after adjusting for age and sex, and, after adjustments, also lower folic acid levels than clinically detected patients (Table 2). On GFD, folic acid and vitamin B12 levels improved significantly in both groups, likewise Hb in clinically detected patients. Screen-detected patients still exhibited higher Hb levels, both in crude analysis and after adjustments, while the other laboratory parameters were comparable between the groups (Table 2).

At diagnosis, 28% of screen-detected and 27% of clinically detected patients had osteoporosis (*P* = 1.000), while respectively 56% and 50% were overweight or obese (*P* =

Table 2. Laboratory Parameters, Bone Density, and Body Mass Index at Celiac Disease Diagnosis and After 1 Year on Treatment in 214 Patients Identified by Screening or in Clinical Practice

Parameters	Screen-detected (n = 102) Median (Q1, Q3)	Clinically detected (n = 112) Median (Q1, Q3)	*P* value[1]	*P* value[2]
Hemoglobin, g/dL				
At diagnosis	140 (132, 149)	128 (121, 136)	**<.001**	**<.001**
On GFD	139 (131, 149)	133 (126, 137)[3]	**<.001**	**.015**
Folic acid, nmol/L				
At diagnosis	389 (268, 491)	409 (316, 500)	.128	**.014**
On GFD	492 (333, 632)[3]	523 (404, 673)[3]	.281	.098
Vitamin B12, pmol/L				
At diagnosis	314 (225, 397)	284 (214, 355)	.328	.228
On GFD	351 (266, 451)[4]	344 (268, 427)[3]	.743	1.000
Lumbar T-score				
At diagnosis	−0.8 (−1.8, 0.5)	−1.2 (−2.0, −0.3)	**.011**	**.004**
On GFD	−0.5 (−1.5, 1.1)[5,6]	−0.9 (−1.9, −0.1)[3,7]	**.017**	**.003**
Femoral T-score				
At diagnosis	−0.7 (−1.6, 0.0)	−1.0 (−1.7, −0.3)	.392	**.025**
On GFD	−0.6 (−1.7, 0.0)[8]	−1.0 (−1.6, −0.2)[3,7]	.420	**.026**
BMI, kg/m^2				
At diagnosis	25.4 (23.0, 28.3)	24.5 (21.1, 33.2)	.585	**.008**
On GFD	25.0 (23.0, 28.1)	24.6 (21.9, 30.8)[9]	.706	**.047**

Screen-detected vs clinically detected patients: [1]crude analysis and [2]adjusted for age and sex. At diagnosis vs on GFD: [3]$P < .001$; [4]$P = .003$; [5]$P = .012$. Data were available for ≥70% of patients, except in [6]68%, [7]58%, [8]67%, and [9]64%.
Bolded *P* value denotes statistical significance.
BMI, body mass index; GFD, gluten-free diet.

.283). Lumbar T-score was higher in screen-detected than in clinically diagnosed patients, both in crude analysis and after adjusting for age and sex. Femoral T-score was also higher after the adjustments (Table 2). On GFD, both groups showed significant improvements in lumbar T-scores and clinically diagnosed patients also in femoral T-score, while there was no significant change in BMI. Lumbar and femoral T-scores were still higher in screen-detected patients after 1 year on GFD (Table 2).

Various symptoms were also reported by 40% of screen-detected patients at diagnosis; however, according to age- and sex-adjusted GSRS scores, these were milder than in clinically detected patients, except for constipation, the severity of which was comparable between the groups (Figure A). All GSRS scores improved in both groups on GFD and, except for slightly lower reflux scores in screen-detected patients, the groups no longer differed in this respect (Figure A).

Overall quality of life was better in screen-detected than in clinically diagnosed patients at diagnosis, also after adjusting for age and sex (median PGWB total score 107 vs 98, $P = .027$). Screen-detected patients had better PGWB-based self-control and general health, whereas the other subscores were comparable between the groups (Figure B). The total score, as well as well-being and vitality subscores, improved in both groups on GFD, whereas the other subscores improved only in the clinically detected patients. There was no longer a significant difference in quality of life between the groups on GFD (Figure B).

In a separate analysis, asymptomatic screen-detected patients (n = 57) exhibited lower lumbar T-score than those reporting symptoms (n = 45), whereas the subgroups did not differ in age, sex, family history for celiac disease, disease serology or histology, Hb levels, BMI, or femoral T-score at diagnosis (Supplementary Table 2). Asymptomatic patients also had lower total GSRS scores and diarrhea, indigestion, and reflux subscores at diagnosis but not on GFD, while all PGWB scores were comparable at both time points. Additionally, adherence to GFD was comparable between the asymptomatic and symptomatic screen-detected patients (Supplementary Table 2).

Discussion

A key finding of this study was that the diagnostic approach was not associated with celiac disease autoantibody levels or with the severity of small-bowel mucosal damage. Furthermore, both screen-detected patients and those diagnosed on a clinical basis benefited from GFD, as evidenced by improvements in laboratory and histologic parameters, BMD, symptom scores, and quality of life. Notably, even apparently asymptomatic patients showed excellent adherence to GFD and reported no decline in quality of life during treatment. Additionally, screening could have identified patients who might not have been recognized in clinical practice due to their health care–seeking behavior or because they did not fit the typical profile of a patient with celiac disease.

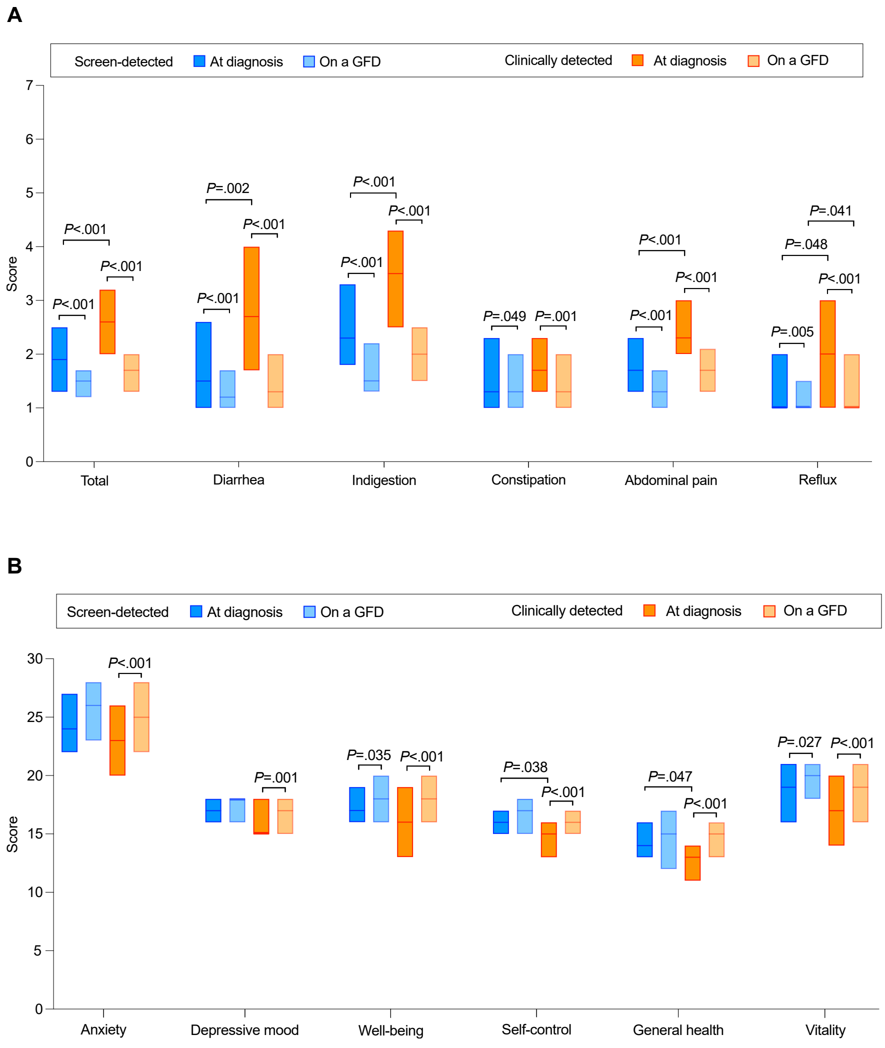

Figure. (A) Comparison of symptoms evaluated by the Gastrointestinal Symptom Rating Scale at celiac disease diagnosis and after 1 year on a gluten-free diet (GFD) between patients identified by screening (n = 102) or in clinical practice (n = 112). Higher scores denote more severe symptoms. Median (horizontal line) and interquartile range (box) are shown. Only significant *P* values after adjustment for age and sex are shown. (B) Comparison of quality of life evaluated by the Psychological General Well-Being questionnaire at celiac disease diagnosis and after 1 year on a gluten-free diet (GFD) between patients identified by screening (n = 102) or in clinical practice (n = 112). Higher scores denote better quality of life. Only subscores are shown. Only significant *P* values after adjustment for age and sex are shown.

In addition to the comparable serologic and histologic markers of celiac disease observed between screen-detected and clinically identified patients, no differences were found between asymptomatic and symptomatic screen-detected patients on these parameters. Regarding mucosal inflammation, screen-detected patients displayed higher total and $\gamma\delta+$ IEL densities, which may be considered unexpected. It is possible that undiagnosed yet symptomatic patients unconsciously reduce their gluten intake, leading to declining disease activity.[24,25] Furthermore, the possible longer duration of untreated inflammation among screen-detected patients could have led to accumulation of more IELs. Screen-detected patients presented with elevated IEL counts even while adhering to GFD. $\gamma\delta+$ IELs may actually play a role in the mucosal repair, and lower densities have even been found to be associated with more severe disease presentation and refractory celiac disease.[26,27] Overall, the role of IELs, particularly $\gamma\delta+$ IELs, remains debated, and further research is needed to clarify their contribution to pathogenesis.[28]

Regarding the association between symptoms and histologic damage, earlier reports have been inconsistent,[14,29–34] which may be partly explained by the use of varying classification systems. Most studies have used grouped histologic categorizations,[30–33] which are prone to intraobserver and interobserver variability,[35–37] although inconsistencies have even been observed in studies using more quantitative measurements, similar to our approach.[14,34] Furthermore, several factors may influence the manifestation of symptoms, including comorbidities, nongluten food components, microbiota-related factors, and individual immune responses.[38–41] Additional complicating factors include temporal trends toward milder disease over time[42] and the potential impact of the length of the histologic lesion on symptoms.[29] The presence of equally advanced mucosal lesion across patients—regardless of the diagnostic approach—could be considered to support active screening to improve underdiagnosis of celiac disease.

It must be emphasized that 40% of our screen-detected patients reported symptoms at diagnosis. Moreover, even among individuals initially considered "asymptomatic," a 1-year GFD led to improved GSRS scores. Although earlier reports on this topic have yielded variable findings,[16,33,43–46] possibly due to differences in study design and methods of

symptom assessment, improvement on GFD has been similarly observed.[16,47,48] On the other hand, as noted, some of the symptoms may be attributable to conditions other than celiac disease, and the benefits of GFD may extend beyond gluten-related mechanisms. Taken together, although defining and objectively measuring symptoms is challenging, "screen-detected" does not necessarily mean "asymptomatic," as unrecognized symptoms of celiac disease appear to be common.

Despite similarly advanced histologic damage, clinically identified patients exhibited lower Hb levels and BMD values, although the prevalence of osteoporosis was similar. For comparison, Tovoli et al[33] reported that osteoporosis was less common in screen-detected than in clinically identified adults with celiac disease at diagnosis, whereas Mustalahti et al[49] found the former to have even more severely impaired BMD. At a general level, screen-detected patients have more frequently had a history of low-energy fractures and lower BMD than nonceliac controls.[43,50] Overrepresentation of iron deficiency and anemia has also previously been observed in screen-detected patients, although, again, less frequently than in those identified on a clinical basis.[33,51] Also, compared to nonceliac controls, screen-detected patients have exhibited lower Hb levels.[52] In line with the present results and supporting the benefits of screening, both BMD and low Hb have improved on GFD.[43,49]

Quality of life and dietary adherence are important considerations when assessing the pros and cons of screening. Asymptomatic patients, in particular, may be at risk for challenges related to these issues. Here, most patients had strict GFD adherence, including those who were asymptomatic, and no differences in quality of life were observed during treatment. This finding is in line with those of several earlier studies,[16,33,44–46,51] although some have reported poorer adherence in screen-detected and/or asymptomatic patients.[16,53,54] Similarly, improvements in quality of life among screen-detected patients have been reported by others,[16,43,45,47,55] although, again, asymptomatic patients may be at risk for poorer outcome.[16,44,51] Various factors may influence the results, such as the availability of GFD products, general awareness of celiac disease,[56] differing study designs, and family history of the disease. Furthermore, the high prevalence of celiac disease and the relative ease in maintaining GFD in Finland may limit the generalizability of our results. Additionally, more long-term studies are needed, as adherence and quality of life may differ from the 'honeymoon period' following diagnosis. Overall, clear communication of the diagnosis and emphasizing the benefits of treatment, especially for asymptomatic patients, are essential, along with identifying those who may require additional support.[57]

Strengths and Limitations

The key strengths of the study include prospective design, the use of validated histologic methods, and standardized questionnaires in assessing quality of life and symptoms. As a limitation, the data on some patients were incomplete, and the collection of comorbidities was nonsystematic. Additionally, classification of patients into screen-detected and clinically found or into symptomatic and asymptomatic groups is always a somewhat arbitrary. For example, a higher-than-expected prevalence of family history of celiac disease was also observed among clinically identified patients, which may reflect greater awareness of the disease and recommendations to screen first-degree relatives once an index case has been diagnosed. Overall, a known family history may result in lower threshold evaluations for celiac disease in relation with various complaints. Additional limitations include self-reported GFD adherence, a relatively short follow-up period for evaluating adherence and quality of life, and potential selection bias in recruitment. Furthermore, although adjusted for age and sex, residual confounding remains possible as group differences in these factors may still have influenced the results. Finally, the results may not be generalizable to settings with lower awareness of celiac disease and limited availability of GFD products.

Conclusion

Celiac disease was histologically similarly advanced regardless of the diagnostic approach or symptom presentation, although the lower prevalence of complications suggests that screening likely led to earlier recognition of the disease. Furthermore, GFD adherence and treatment response—including BMD, gastrointestinal symptoms, and quality of life—were comparable across patient groups, even among those with asymptomatic presentation, supporting the benefits of diagnosing these patients. Our results provide additional support for systematic screening for celiac disease, at least in countries with high awareness of the condition and easy access to gluten-free products. The findings also call into question whether dividing patients into groups based on clinical phenotype or diagnostic approach is meaningful, as these factors do not appear to reflect biologically distinct disease. At the same time, however, more evidence is needed on the long-term outcomes of screen-detected, particularly asymptomatic, celiac disease.

Supplementary Materials

Material associated with this article can be found, in the online version, at https://doi:10.1016/j.gastha.2026.100973.

References

1. Lindfors K, Ciacci C, Kurppa K, et al. Coeliac disease. Nat Rev Dis Primers 2019;5:3.
2. Taavela J, Kurppa K, Jääskeläinen T, et al. Trends in the prevalence rates and predictive factors of coeliac disease: a long-term nationwide follow-up study. Aliment Pharmacol Ther 2024;59:372–379.

3. Singh P, Arora A, Strand TA, et al. Global prevalence of celiac disease: systematic review and meta-analysis. Clin Gastroenterol Hepatol 2018;16:823–836.e2.
4. Fuchs V, Kurppa K, Huhtala H, et al. Factors associated with long diagnostic delay in celiac disease. Scand J Gastroenterol 2014;49:1304–1310.
5. Stahl MG, Geno Rasmussen C, Dong F, et al. Mass screening for celiac disease: the autoimmunity screening for kids study. Am J Gastroenterol 2021; 116:180–187.
6. Ludvigsson JF, Bai JC, Biagi F, et al. Diagnosis and management of adult coeliac disease: guidelines from the British Society of Gastroenterology. Gut 2014; 63:1210–1228.
7. Al-Toma A, Volta U, Auricchio R, et al. European Society for the Study of Coeliac Disease (ESsCD) guideline for coeliac disease and other gluten-related disorders. United European Gastroenterol J 2019;7:583–613.
8. Rubio-Tapia A, Hill ID, Semrad C, et al. American College of Gastroenterology guidelines update: diagnosis and management of celiac disease. Am J Gastroenterol 2023;118:59–76.
9. Husby S, Koletzko S, Korponay-Szabó I, et al. European Society Paediatric Gastroenterology, Hepatology and Nutrition guidelines for diagnosing coeliac disease 2020. J Pediatr Gastroenterol Nutr 2020;70:141–156.
10. Bosi E, Catassi C. Screening type 1 diabetes and celiac disease by law. Lancet Diabetes Endocrinol 2024; 12:12–14.
11. Chou R, Bougatsos C, Blazina I, et al. Screening for celiac disease: evidence report and systematic review for the US Preventive Services Task Force. JAMA 2017; 317:1258–1268.
12. Ludvigsson JF, Card TR, Kaukinen K, et al. Screening for celiac disease in the general population and in high-risk groups. United European Gastroenterol J 2015; 3:106–120.
13. Laurikka P, Kivelä L, Kurppa K, et al. Review article: systemic consequences of coeliac disease. Aliment Pharmacol Ther 2022;56 Suppl 1(Suppl 1):S64–S72.
14. Taavela J, Kurppa K, Collin P, et al. Degree of damage to the small bowel and serum antibody titers correlate with clinical presentation of patients with celiac disease. Clin Gastroenterol Hepatol 2013;11:166–171.e1.
15. See JA, Kaukinen K, Makharia GK, et al. Practical insights into gluten-free diets. Nat Rev Gastroenterol Hepatol 2015;12:580–591.
16. Ukkola A, Mäki M, Kurppa K, et al. Diet improves perception of health and well-being in symptomatic, but not asymptomatic, patients with celiac disease. Clin Gastroenterol Hepatol 2011;9:118–123.
17. Sulkanen S, Collin P, Laurila K, et al. IgA- and IgG-class antihuman umbilical cord antibody tests in adult coeliac disease. Scand J Gastroenterol 1998;33:251–254.
18. Hill PG, Forsyth JM, Semeraro D, et al. IgA antibodies to human tissue transglutaminase: audit of routine practice confirms high diagnostic accuracy. Scand J Gastroenterol 2004;39:1078–1082.
19. Oberhuber G, Granditsch G, Vogelsang H. The histopathology of coeliac disease: time for a standardized report scheme for pathologists. Eur J Gastroenterol Hepatol 1999;11:1185–1194.
20. Järvinen TT, Kaukinen K, Laurila K, et al. Intraepithelial lymphocytes in celiac disease. Am J Gastroenterol 2003; 98:1332–1337.
21. Sözen T, Özışık L, Başaran NÇ. An overview and management of osteoporosis. Eur J Rheumatol 2017; 4:46–56.
22. Svedlund J, Sjödin I, Dotevall G. GSRS - a clinical rating scale for gastrointestinal symptoms in patients with irritable bowel syndrome and peptic ulcer disease. Dig Dis Sci 1988;33:129–134.
23. Dimenäs E, Carlsson G, Glise H, et al. Relevance of norm values as part of the documentation of quality of life instruments for use in upper gastrointestinal disease. Scand J Gastroenterol Suppl 1996;221:8–13.
24. Sánchez-Castañon M, Castro BG, Toca M, et al. Intraepithelial lymphocytes subsets in different forms of celiac disease. Auto Immun Highlights 2016;7:14.
25. García-Hoz C, Crespo L, Pariente R, et al. Intraepithelial lymphogram in the diagnosis of celiac disease in adult patients: a validation cohort. Nutrients 2024;16:1117.
26. Saukkonen J, Kaukinen K, Koivisto AM, et al. Clinical characteristics and the dietary response in celiac disease patients presenting with or without anemia. J Clin Gastroenterol 2017;51:412–416.
27. Verbeek WHM, von Blomberg BME, Scholten PET, et al. The presence of small intestinal intraepithelial gamma/delta T-lymphocytes is inversely correlated with lymphoma development in refractory celiac disease. Am J Gastroenterol 2008;103:3152–3158.
28. Kang I, Kim Y, Lee HK. Double-edged sword: $\gamma\delta$ T cells in mucosal homeostasis and disease. Exp Mol Med 2023;55:1895–1904.
29. Murray JA, Rubio-Tapia A, Van Dyke CT, et al. Mucosal atrophy in celiac disease: extent of involvement, correlation with clinical presentation, and response to treatment. Clin Gastroenterol Hepatol 2008;6:186–193, quiz 125.
30. Brar P, Kwon GY, Egbuna II, et al. Lack of correlation of degree of villous atrophy with severity of clinical presentation of coeliac disease. Dig Liver Dis 2007; 39:26–29, discussion 30–32.
31. Thomas HJ, Ahmad T, Rajaguru C, et al. Contribution of histological, serological, and genetic factors to the clinical heterogeneity of adult-onset coeliac disease. Scand J Gastroenterol 2009;44:1076–1083.
32. Rubio-Tapia A, Van Dyke CT, Lahr BD, et al. Predictors of family risk for celiac disease: a population-based study. Clin Gastroenterol Hepatol 2008;6:983–987.
33. Tovoli F, Negrini G, Sansone V, et al. Celiac disease diagnosed through screening programs in at-risk adults is not associated with worse adherence to the gluten-free diet and might protect from osteopenia/osteoporosis. Nutrients 2018;10:1940.
34. Käräjämäki AJ, Taavela J, Nielsen C, et al. Celiac disease antibody levels reflect duodenal mucosal damage but not clinical symptoms. Scand J Gastroenterol 2021; 56:514–519.
35. Werkstetter KJ, Korponay-Szabó IR, Popp A, et al. Accuracy in diagnosis of celiac disease without biopsies in clinical practice. Gastroenterology 2017;153:924–935.
36. Mubarak A, Nikkels P, Houwen R, et al. Reproducibility of the histological diagnosis of celiac disease. Scand J Gastroenterol 2011;46:1065–1073.

37. Ravelli A, Villanacci V, Monfredini C, et al. How patchy is patchy villous atrophy?: distribution pattern of histological lesions in the duodenum of children with celiac disease. Am J Gastroenterol 2010;105:2103–2110.
38. Caio G, Volta U, Sapone A, et al. Celiac disease: a comprehensive current review. BMC Med 2019;17:142.
39. Wacklin P, Laurikka P, Lindfors K, et al. Altered duodenal microbiota composition in celiac disease patients suffering from persistent symptoms on a long-term gluten-free diet. Am J Gastroenterol 2014;109:1933–1941.
40. Mehta S, Agarwal A, Pachisia AV, et al. Impact of delay in the diagnosis on the severity of celiac disease. J Gastroenterol Hepatol 2024;39:256–263.
41. Laurikka P, Kaukinen K, Kurppa K. Unravelling the mechanisms behind the persistent gastrointestinal symptoms in celiac disease - how can they lead to better treatment outcomes? Expert Rev Gastroenterol Hepatol 2017;11:605–607.
42. Kivelä L, Kaukinen K, Lähdeaho ML, et al. Presentation of celiac disease in finnish children is no longer changing: a 50-year perspective. J Pediatr 2015; 167:1109–1115.e1.
43. Vilppula A, Kaukinen K, Luostarinen L, et al. Clinical benefit of gluten-free diet in screen-detected older celiac disease patients. BMC Gastroenterol 2011;11:136.
44. Mahadev S, Gardner R, Lewis SK, et al. Quality of life in screen-detected celiac disease patients in the United States. J Clin Gastroenterol 2016;50:393–397.
45. Mustalahti K, Lohiniemi S, Collin P, et al. Gluten-free diet and quality of life in patients with screen-detected celiac disease. Eff Clin Pract 2002;5:105–113.
46. Paavola A, Kurppa K, Ukkola A, et al. Gastrointestinal symptoms and quality of life in screen-detected celiac disease. Dig Liver Dis 2012;44:814–818.
47. Kinos S, Kurppa K, Ukkola A, et al. Burden of illness in screen-detected children with celiac disease and their families. J Pediatr Gastroenterol Nutr 2012;55:412–416.
48. Kurppa K, Paavola A, Collin P, et al. Benefits of a gluten-free diet for asymptomatic patients with serologic markers of celiac disease. Gastroenterology 2014;147:610–617.e1.
49. Mustalahti K, Collin P, Sievänen H, et al. Osteopenia in patients with clinically silent coeliac disease warrants screening. Lancet 1999;354:744–745.
50. Björck S, Brundin C, Karlsson M, et al. Reduced bone mineral density in children with screening-detected celiac disease. J Pediatr Gastroenterol Nutr 2017;65:526–532.
51. Kivelä L, Popp A, Arvola T, et al. Long-term health and treatment outcomes in adult coeliac disease patients diagnosed by screening in childhood. United European Gastroenterol J 2018;6:1022–1031.
52. Al-Hussaini A, Troncone R, Alobaid S, et al. Status of vitamins and minerals in children with screening-identified celiac disease: a case-control study. J Pediatr Gastroenterol Nutr 2024;78:677–684.
53. Cozzi G, Gabbana E, Zanchi C, et al. 20-Year follow-up study of celiac patients identified in a mass school screening: compliance to gluten-free diet and autoimmunity. J Pediatr Gastroenterol Nutr 2022;74:91–95.
54. Fabiani E, Taccari LM, Rätsch IM, et al. Compliance with gluten-free diet in adolescents with screening-detected celiac disease: a 5-year follow-up study. J Pediatr 2000;136:841–843.
55. Kvamme JM, Sørbye S, Florholmen J, et al. Population-based screening for celiac disease reveals that the majority of patients are undiagnosed and improve on a gluten-free diet. Sci Rep 2022;12:12647.
56. White LE, Bannerman E, Gillett PM. Coeliac disease and the gluten-free diet: a review of the burdens; factors associated with adherence and impact on health-related quality of life, with specific focus on adolescence. J Hum Nutr Diet 2016;29:593–606.
57. Kurppa K, Mulder CJ, Stordal K, et al. Celiac disease affects 1% of global population: who will manage all these patients? Gastroenterology 2024;167:148–158.

Received February 19, 2026. Accepted April 10, 2026.

Correspondence:
Address correspondence to: Kalle Kurppa, MD, PhD, Celiac Disease Research Center, Tampere University, Arvo Building, Arvo Ylpön katu 34, Tampere 33520, Finland. e-mail: kalle.kurppa@tuni.fi.

Authors' Contributions:
Iida Ahonen: Formal analysis, investigation, methodology, visualization, writing - original draft. Laura Kivelä: Funding acquisition, methodology, Project administration, supervision, validation, writing - review & editing. Aku Paavola: Methodology, writing - review & editing. Pilvi Laurikka: Writing - review & editing. Heini Huhtala: Formal analysis, writing - review & editing. Harri Sievänen: Data curation, writing - review & editing. Katri Kaukinen: Conceptualization, data curation, funding acquisition, methodology, resources, Validation, Writing - review & editing. Kalle Kurppa: Conceptualization, data curation, funding acquisition, methodology, project administration, Resources, supervision, validation, writing - review & editing.

Conflicts of Interest:
These authors disclose the following: Laura Kivelä has received travel grant from the International Society for the Study of Celiac Disease and Takeda. K.Ku. has received consultation and/or lecture fees from Takeda, Thermo Fisher, and Sanofi. The remaining authors disclose no conflicts.

Funding:
This study was supported by the Foundation for Pediatric Research, the Competitive State Research Financing of the Expert Area of Tampere University Hospital, the Päivikki and Sakari Sohlberg Foundation, the Maire Rossi Foundation, the Emil Aaltonen Foundation, the Sigrid Juselius Foundation, the Research Council of Finland, the Finnish Medical Foundation, and the Finnish Celiac Society.

Ethical Statement:
The Regional Ethics Committees of Tampere University Hospital and the Pirkanmaa Hospital District approved the study protocol and patient recruitment (ethical committee codes E98012, R03041, and R07122). All participants were informed about the study and provided written informed consent. They were aware of their option to withdraw their consent to participate at any time without this affecting their treatment. The ethical guidelines of the Declaration of Helsinki were strictly followed.

Data Transparency Statement:
Due to privacy and confidentiality concerns, the data supporting the findings of this study are not publicly available. De-identified data are available from the corresponding author upon reasonable request.

Reporting Guidelines:
STROBE.

Gastro Hep Advances, Volume *5*

Supplemental information

Clinical, Serologic, and Histologic Characteristics in Screen-Detected and Clinically Diagnosed Celiac Disease Patients

Iida Ahonen, Laura Kivelä, Aku Paavola, Pilvi Laurikka, Heini Huhtala, Harri Sievänen, Katri Kaukinen, and Kalle Kurppa

Supplementary Table 1. Presence of co-morbidities in 214 celiac disease patients identified by screening or in clinical practice.

	Screen-detected n=102 %	Symptom-detected n=112 %	*P* value
Asthma	6	6	1.000
Coronary artery disease	5	5	1.000
Hypertension	16	10	.303
Lactose intolerance	11	11	1.000
Malignancy[1]	6	4	.529
Neurological disorder[2]	4	1	.202
Other autoimmune disease[3]	8	4	.242
Psoriasis	5	1	.111
Psychiatric disorder[4]	5	6	1.000
Thyroidal disease	15	18	.581
Type 1 diabetes	3	2	.676

[1]Breast cancer, lymphoma, adenocarcinoma, prostate carcinoma, ovarian carcinoma, ventricular carcinoma, basal cell carcinoma, melanoma; [2]Hemiplegia, polyneuropathy, benign paroxysmal positional vertigo, cerebral infarction; [3]Sjögren's syndrome, IgA nephropathy, IgA deficiency, systemic lupus erythematosus, alopecia, rheumatoid arthritis; [4]Depression, panic disorder, obsessive-compulsive disorder, vital exhaustion.

Supplementary Table 2. Clinical characteristics at diagnosis, and symptoms and health-related quality of life at diagnosis and after one year of treatment in 102 screen-detected celiac disease patients with or without self-reported symptoms at diagnosis.

	Asymptomatic n=57	Symptomatic n=45	*P* value[1]
	%	%	
Females	47	53	.690
Family history of celiac disease	67	74	.511
Strict GFD adherence	91	98	.225
	Median (Q1, Q3)	Median (Q1, Q3)	
Age, years	53 (37, 62)	54 (43, 59)	.701
TGA, U/mL	18.3 (7.3, 47.7)	28.6 (11.3, 76.0)	.263
EmA, titer	1:200 (1:50, 1:500)	1:200 (1:50, 1:500)	.681
VH/CrD	0.3 (0.1, 0.9)	0.2 (0.1, 0.8)	.524
IELs, /100 epithelial cells	52.1 (37.8, 64.5)	49.8 (35.4, 65.2)	.949
Hemoglobin, g/dL	140 (132, 148)	140 (131, 153)	.363
Lumbar T-score	-1.0 (-2.0, 0.0)	-0.4 (-1.4, 1.2)	**.018**
Femoral T-score	-0.9 (-1.9, -0.1)	-0.6 (-1.5, 0.0)	.287
BMI, kg/m^2	24.6 (22.4, 28.0)	24.3 (22.7, 28.5)	.066
GSRS, total score			
At diagnosis	1.6 (1.3, 2.4)	2.2 (1.6, 2.6)	**.011**
On GFD	1.4 (1.1, 1.7)[1]	1.6 (1.2, 1.8)[1]	.080
PGWB, total score[2]			
At diagnosis	108 (101, 117)	107 (89, 113)	.305
On GFD	113 (105, 119)[3]	110 (100, 119)	.312

[1]Significant improvement on GFD, *P*<.001. [2]No significant differences in sub-scores between the groups at diagnosis or on GFD. [3]Significant improvement on a GFD, *P*=.015. BMI, body mass index; EmA, endomysial antibodies; GSRS, Gastrointestinal Symptom Rating Scale, higher scores denote more symptoms; IELs, intraepithelial lymphocytes; PGWB, Psychological General Well-Being questionnaire, higher scores denote better quality of life; TGA, transglutaminase 2 antibodies; VH/CrD, villous height-crypt depth ratio.
